# Non-Uniform and Non-Random Binding of Nucleoprotein to Influenza A and B Viral RNA

**DOI:** 10.3390/v10100522

**Published:** 2018-09-25

**Authors:** Valerie Le Sage, Adalena V. Nanni, Amar R. Bhagwat, Dan J. Snyder, Vaughn S. Cooper, Seema S. Lakdawala, Nara Lee

**Affiliations:** 1 Department of Microbiology and Molecular Genetics, University of Pittsburgh School of Medicine, Pittsburgh, PA 15217, USA; valerie.lesage@pitt.edu (V.L.S.); avnanni@gmail.com (A.V.N.); abhagwat@pitt.edu (A.R.B.); djs150@pitt.edu (D.J.S.); vaughn.cooper@pitt.edu (V.S.C.); 2 Center for Evolutionary Biology and Medicine, University of Pittsburgh School of Medicine, Pittsburgh, PA 15217, USA

**Keywords:** influenza virus, nucleoprotein, viral RNA, HITS-CLIP, viral ribonucleoprotein

## Abstract

The genomes of influenza A and B viruses have eight, single-stranded RNA segments that exist in the form of a viral ribonucleoprotein complex in association with nucleoprotein (NP) and an RNA-dependent RNA polymerase complex. We previously used high-throughput RNA sequencing coupled with crosslinking immunoprecipitation (HITS-CLIP) to examine where NP binds to the viral RNA (vRNA) and demonstrated for two H1N1 strains that NP binds vRNA in a non-uniform, non-random manner. In this study, we expand on those initial observations and describe the NP-vRNA binding profile for a seasonal H3N2 and influenza B virus. We show that, similar to H1N1 strains, NP binds vRNA in a non-uniform and non-random manner. Each viral gene segment has a unique NP binding profile with areas that are enriched for NP association as well as free of NP-binding. Interestingly, NP-vRNA binding profiles have some conservation between influenza A viruses, H1N1 and H3N2, but no correlation was observed between influenza A and B viruses. Our study demonstrates the conserved nature of non-uniform NP binding within influenza viruses. Mapping of the NP-bound vRNA segments provides information on the flexible NP regions that may be involved in facilitating assembly.

## 1. Introduction

Influenza viruses cause worldwide outbreaks of mild to severe respiratory disease and is a constant public health concern each year. They are genetically diverse and are divided into four types A, B, C, and D, which differ in their genetic organization, host species, and clinical-epidemiological characteristics. During seasonal epidemics, influenza B viruses (IBV), influenza A/H3N2, and A/H1N1 viruses are continuously co-circulating and cause a significant disease burden [1]. Influenza A virus (IAV) has a broad species tropism while IBV exists predominantly within the human population [2].

The single-stranded, negative-sense viral RNA (vRNA) genome comprises eight segments, which are each named for the major protein that it encodes (PB2, PB1, PA, HA, NP, NA, M, NS) and vary in length from 2.3 kb to 0.9 kb. Packaging of all eight vRNA segments is required for production of fully infectious virus particles [3,4] and occurs en route to the plasma membrane [5,6]. Evidence suggests that assembly is a selective process [7,8,9], which results in a distinct pattern of seven vRNA in a circle surrounding a single one in the center [10,11,12,13]. The precise mechanism of selective assembly is still unknown, but direct RNA-RNA interactions have been proposed to mediate formation of the vRNA supra-molecule [10,14].

Each influenza vRNA is bound by multiple nucleoprotein (NP) molecules and one heterotrimeric RNA polymerase complex composed of PB2, PB1, and PA to form a viral ribonucleoprotein (vRNP) complex. NP is the most abundant protein in the vRNP complex and provides a scaffold for the vRNA to facilitate the formation of the double helical vRNP structure, which results from NP binding RNA and self-oligomerization [15,16,17]. Prevailing schematics of the viral genome depict a uniform string of NP along the length of the vRNA segment. However, this classical architecture would make segment-specific RNA-RNA interactions difficult to form. Given the fact that the classical viral genomic structure was constructed from EM images [18,19], which provides no information regarding the RNA structure, we proposed that NP could associate with vRNA in a non-random and non-uniform manner. We previously used high-throughput RNA sequencing coupled with crosslinking immunoprecipitation (HITS-CLIP) to map NP association to vRNA for all eight segments of two H1N1 strains [20] and found that NP bound to RNA in a non-uniform, non-random manner. This was highly reproducible. Recently, another group using a similar technique (PAR-CLIP) also found that NP does not bind randomly and uniformly to H1N1 vRNA [21]. These studies propose a new architecture of the influenza viral genome.

In this study, we performed HITS-CLIP to map NP association to vRNA for all eight segments of a seasonal H3N2 and an IBV strain. These viruses were chosen since they co-circulate with seasonal H1N1 viruses and have the potential to reassort in nature. We demonstrate that H3N2 has some correlation to the NP-vRNA binding profiles of H1N1 while IAV and IBV strains have no correlation in their NP-binding profiles. Our studies demonstrate that the non-uniform, non-random NP-binding pattern is consistent for all human seasonal viruses and is a conserved feature of the influenza genomic architecture.

## 2. Materials and Methods

### 2.1. Cells and Viruses

Madin-Darby canine kidney (MDCK) cells were maintained in Eagle’s Minimum Essential Medium (EMEM, Sigma-Aldrich, St. Louis, MO, USA) supplemented with 10% fetal bovine serum (FBS, Hyclone, Logan, UT, USA), 2 mM L-glutamine (Gibco, Waltham, MA, USA), and 1% penicillin/streptomycin (Gibco). A/Panama/07/1999 (H3N2) virus was a generous gift from Dr. Zhiping Ye (Center for Biologics Evaluation and Research, FDA) through the WHO Global Influenza Surveillance Response System. The influenza B virus, B/Texas/02/2013 (Victoria Lineage), Cell-Derived, FR-1302, was obtained through the Influenza Reagent Resource, Influenza Division, WHO Collaborating Center for Surveillance, Epidemiology and Control of Influenza, Centers for Disease Control and Prevention, Atlanta, GA, USA.

### 2.2. HITS-CLIP and Deep Sequencing Data Analysis

HITS-CLIP was performed as described previously [20]. Two confluent T175 flasks of MDCK cells were washed with phosphate-buffered saline and infected with the indicated virus at a dilution of 1:10,000 in serum-free EMEM containing TPCK-trypsin (Worthington Biochemicals, Berkshire, UK). At 96 h post-infection, 40 mL of the culture medium containing ~10^7^ infectious virus particles per mL was harvested and cellular debris was pelleted by centrifugation at 2000× *g* for 20 min. A clarified supernatant was UV irradiated at 254 nm (400 mJ/cm^2^ followed by 200 mJ/cm^2^) and ultra-centrifuged at 200,000× *g* for 2 h on a 30% sucrose-NTE (100 mM NaCl, 10 mM Tris pH 7.4, 1 mM EDTA) cushion. Concentrated virions were re-suspended in PXL buffer (1× PBS, 1% NP40, 0.5% deoxycholate, 0.1% SDS), treated with DNase and RNase, and it was followed by a partial RNase A (0.25, 0.025, and 0.0025 μg) digestion for 5 min at 37 °C. Virus lysate was incubated with the Dynabead Protein G conjugated to mouse monoclonal antibody MAB8251 (Millipore, Burlington, MA, USA) for A/Panama/07/1999 or mouse monoclonal antibody ab2074 (Abcam, Cambridge, MA, USA) for B/Texas/02/2013 to immuno-precipitate influenza NP. 5′ and 3′ adaptor ligation, RT reaction, and first-round PCR amplification were performed before preparing Illumina-compatible deep sequencing libraries using the NEBNext Ultra DNA Library Prep Kit (NEB, Ipswich, MA, USA). Deep sequencing was carried out using Illumina’s NextSeq platform and the data were deposited in the Sequence Read Archive under accession numbers SRP158158. Data analysis was performed using the NovoAlign alignment program and mapping the reads to reference genomes available from the NCBI database. Sequencing results were visualized on the Integrated Genome Viewer [22].

### 2.3. Pearson Correlation Analysis

HITS-CLIP data were normalized to the highest peak within each segment (excluding super peaks). The normalized read depth at each nucleotide position was compared between H1N1, H3N2, and IBV strains using the Prism 6 software (GraphPad, La Jolla, CA, USA). The Pearson correlation coefficients and corresponding *p*-values were determined between influenza virus strains for each segment. In general, Pearson correlation coefficients (*r*) range from 1 to −1 where *r* ≥ 0.7 demonstrates a high positive correlation, 0.5 ≤ *r* < 0.7 is a moderate positive correlation, 0.3 ≤ *r* < 0.5 is a low positive correlation, and *r* < 0.3 is a negligible correlation [23]. To compare IAV and IBV vRNA segments with different lengths, the sequences of the two strains were aligned using a multiple sequence aligner and the gap corrected by padding any gaps with the preceding values in the bedGraph file.

### 2.4. Peak Analysis

Predicted NP binding sites were determined using the peak-finding algorithm MACS [24]. For each HITS-CLIP experiment, a p-score was chosen that exhibited the best performance in terms of calling NP peaks. Non-peak regions were anything not called a peak by MACS and include HITS-CLIP read coverage of less than 5% of the maximum peak height of each experiment. Mean peak widths were calculated using the coordinates obtained from MACS analysis and omitting apparent double peaks that were called a single peak by the algorithm.

Nucleotide composition for peaks was determined using the coordinates provided by the MACS software. The sequences were isolated and the percentages of A, U, G, and C were calculated based on the length of the peak. Nucleotide compositions of non-peak regions were calculated in the same manner. The nucleotide percentages for each peak and non-peak were graphed as a scatter plot to illustrate the observed variation. A two-way ANOVA analysis using Prism 6 software (GraphPad) identified a highly significant difference in the G and U content between peaks and non-peaks (*p*-value < 0.0001). No statistically significant difference was observed between the percentage of A and C bases.

### 2.5. Continuous Wavelet Transforms

To examine potential periodicity in the NP-vRNA binding patterns, continuous one-dimensional wavelet transforms (CWT) were performed on NP-vRNA binding profiles using the “Analytical Morlet” wavelet package within the MATLAB wavelet toolbox. The sampling rate was defined as 1 per nucleotide and the resulting wavelet analysis was mapped using filled contour plots with a nucleotide repetition length as the “y-axis” frequency parameter.

## 3. Results

### 3.1. Seasonal H3N2 and IBV NP-vRNA Binding Profiles

**Figure 1:**
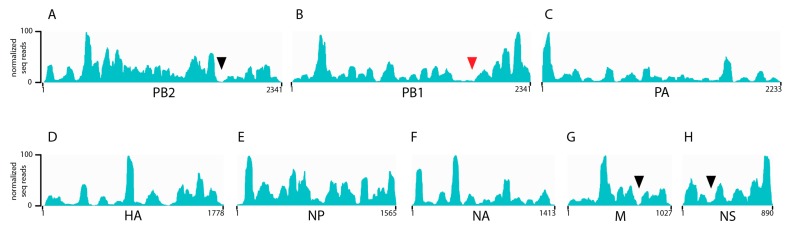
NP binding profile for seasonal H3N2 A/Panama/07/1999 strain (**A**–**H**). The abundance of NP on each vRNA segment was examined using HITS-CLIP on purified virions. Cell-grown virions were concentrated on a sucrose cushion, UV-crosslinked, treated with a partial RNase digestion, and was followed by immunoprecipitation with an anti-NP monoclonal antibody. A representative profile for each segment is presented as an IGV track. The nucleotide lengths and segment names are shown on the x-axis. Normalized reads, based on the highest peak, are displayed on the y-axis. Low NP-binding areas are indicated by black and red arrowheads.

**Figure 2:**
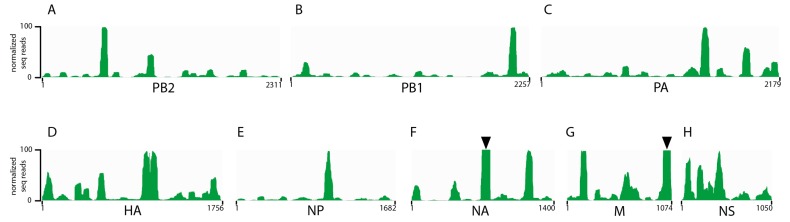
The NP binding profile for the IBV B/Texas/02/2013 strain (**A**–**H**). The abundance of NP on each vRNA segment was examined using HITS-CLIP analysis on purified virions with an anti-IBV NP specific antibody. A representative profile for each segment is presented as an IGV track. The nucleotide lengths and segment names are shown on the x-axis. Normalized reads based on the highest peak excluding “super peaks” (black arrowheads) are displayed on the y-axis.

Previous studies by us and others examining the architecture of the influenza genome using cross-linking immunoprecipitation based approaches have been performed exclusively on H1N1 influenza A strains [20,21]. To determine whether the non-uniform, non-random binding pattern of NP on viral RNA was conserved to other human seasonal influenza viruses. NP HITS-CLIP was performed on a purified seasonal H3N2 and IBV viruses generated from MDCK cells infected with A/Panama/07/1999 (H3N2) or an IBV strain of the Victoria lineage (B/Texas/02/2013). As with H1N1 viruses [20], the H3N2 NP association differed throughout the genome, which demonstrates enriched binding of NP to certain vRNA regions (Figure 1). Interestingly, very few NP-free regions were observed in each of the H3N2 segments with segment 2 (*PB2*), 7 (*M*), and 8 (*NS*) each containing only one such region (Figure 1, black arrowheads). Similar to IAV, we observed a non-uniform and non-random binding profile of NP association for IBV vRNA (Figure 2), which demonstrates that this architecture of the influenza viral genome is conserved between IAV and IBV. In addition, we observed a few areas of specific vRNA segments that were so abundant in our HITS-CLIP that we termed them ‘super peaks’ (Figure 2, black arrowheads). These were seen in IBV segment 6 (*NA*) as well as in IBV segment 7 (*M*). The significance of these highly enriched NP sites is unclear and requires further exploration. Overall, the genomic architecture of H1N1 strains where NP binds to viral RNA in a non-uniform non-random manner is consistent in seasonal H3N2 and IBV viruses.

### 3.2. Characterization of Enriched NP-Binding Regions

We performed a number of analyses on the peaks from all the IAV and IBV strains to understand whether there are any common properties that elicit NP binding. First, we parsed out peaks versus non-peaks for each segment from both strains by using the peak calling algorithm MACS [24]. The sequence for all peaks was then analyzed using a motif finder software, MEME [25,26], but no common sequence motifs were observed.

**Figure 3:**
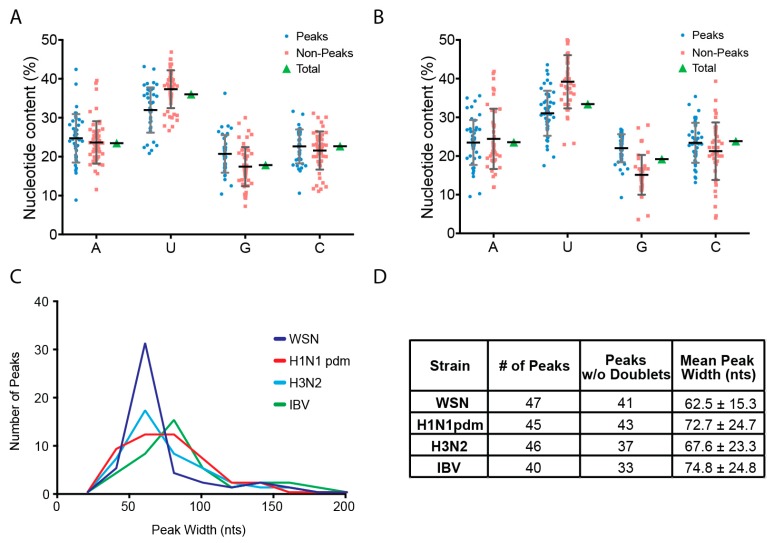
Characterization of NP-peaks. Regions of the vRNA segment that correspond to NP-peaks were identified using the peak calling algorithm MACS [24]. The percentage of each nucleotide was calculated for each peak and compared to non-peak regions as well as to the total genome sequence for A/Panama/07/99 H3N2 (**A**) and B/Texas/02/2013 (**B**). This analysis identified a statistically significant nucleotide bias for NP association in peaks, which are G-rich and U-depleted compared to non-peaks. (**C**) Histogram of the nucleotide foot-print of peaks from four strains: WSN, A/CA/07/09 H1N1pdm, A/Panama/07/99 H3N2, and B/Texas/02/2013. (**D**) Summary of the peak number and width described in C.

We have shown previously that NP from two H1N1 strains preferentially bound vRNA that is G-rich and U-poor compared to non-peaks [20]. To examine whether the same nucleotide bias is present in the IAV and IBV strains analyzed in this study, we calculated the nucleotide content of peak and non-peak regions and compared them to each other as well as the overall genome-wide content (Figure 3A,B). For seasonal H3N2, peaks were significantly enriched in guanine (G) residues when compared to the non-peak content (22% compared to 15%) while uracil (U) bases were relatively depleted (31% compared to 39%) (Figure 3A). This observation was also true for IBV with peaks having 21% G bases as compared to 17% in non-peaks and U bases being present at 32% in peaks and 37% in non-peaks (Figure 3B). These differences were statistically significant based on a two-way ANOVA analysis (*p*-value < 0.0001). The content of A and C bases were maintained at approximately 23% in both populations. Taken together, these data suggest that both IAV and IBV NP proteins preferably associate with relatively G-rich and U-poor regions of vRNA.

Next, we examined the nucleotide width of NP peaks. As depicted in Figure 3C,D, we observed that WSN and H3N2 had similar NP peak widths while they were slightly wider in H1N1pdm. Intriguingly, the peak width of IBV is shifted to include even more nucleotides than IAV NP peaks, which suggests a larger footprint of IBV NP compared to IAV NP. IBV NP has an additional 50 amino acids in the N-terminus [27] and the increased peak width may reflect the larger size of the NP protein. The RNA binding pocket of each NP monomer can associate with 20 to 26 nucleotides [19,28,29]. Therefore, the widths of the HITS-CLIP peaks for all segments of IAV and IBV suggests that a peak consists of approximately three to four NP proteins. Consistent with our results, structural studies have crystalized IAV NP as trimeric complexes [30,31] while IBV NP has been crystalized as tetramers [32]. The common peak width in IAV capable of comprising an NP trimer may suggest a unique property of the NP oligomer assembly that contributes to vRNA association rather than being an artifact of crystal formation.

**Figure 4:**
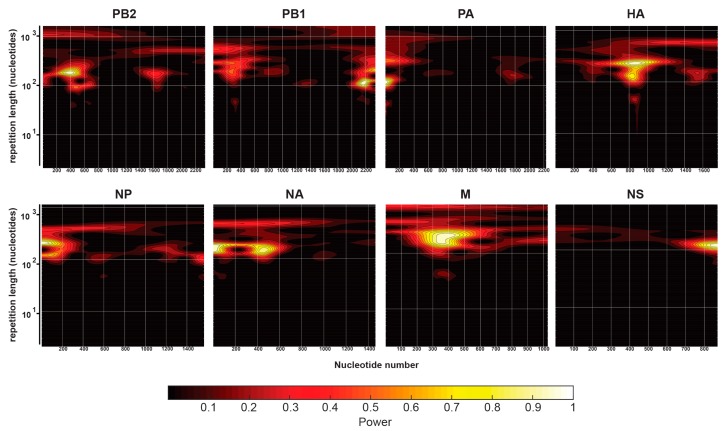
Normalized wavelet transforms of NP-vRNA binding profiles for each seasonal H3N2 A/Panama/07/1999 segment. Binding profiles were analyzed for patterns that could originate from NP-vRNA interaction and occur at regular intervals with the NP scaffold structure. Regular NP contact over the entire length of the NP vRNA segment should show up as a strong horizontal smear (yellow-white) across the graph, which is centered vertically at the repetition interval. The signal of the wavelets power is color coded, according to the scale bar. Each graph is presented as the x-axis: location along the segment, y-axis: interval over which binding is repeated. Repetition lengths are meaningful for a repetition length under 100 nucleotides because a signal at long repetition lengths of the order of the segment length could involve chance contact at 2 to 3 locations only.

Lastly, we explored whether a consistent underlying pattern in NP-vRNA binding could be identified. Specifically, the possible existence of a hidden periodicity to the NP-vRNA binding pattern extends over the entire length of the vRNA for each strain. We performed a periodicity analysis on NP-vRNA binding profiles of all segments for each of the influenza strains using continuous wavelet transformations (CWT). CWT provides a visually intuitive way to analyze the periodicity of the entire binding profile but only a small section at a time, which is similar to a local Fourier analysis. The resulting CWT plots for the seasonal H3N2 are shown in Figure 4 for all eight segments. The two-dimensional contour plots in Figure 4 display the repetition interval on the y-axis, nucleotide position on the x-axis, and the magnitude of periodicity is shown as color-coded signal intensity at a given position. A long-range periodicity, if present, would be visible as a band of signal, horizontal to the x-axis, across the entire CWT contour plot. The only signals that were observed across the entire CWT contour plot were those at repeat lengths of 1000, which would be consistent of peaks found roughly at each end of the genome. Therefore, based on the CWT analysis, no clear periodicity of NP binding along the length of the viral genome was observed for any segment of seasonal H3N2 (Figure 4). Additionally, CWT analysis was performed for all four strains and we observed that the CWT for each segment was strain specific and varied between segments for a given viral strain. These data confirm that NP-vRNA binding profiles are non-uniform and non-periodic.

### 3.3. Comparison of IAV and IBV NP-vRNA Binding Profile

To determine if the NP binding profiles for a given segment were conserved across strains, we calculated the Pearson correlation coefficient for each segment and, as a whole genome, where all eight segments were concatenated together between all four strains. Pearson correlations provide a quantitative assessment of the relatedness of two patterns where *r* ≥ 0.7 indicates a high positive correlation, 0.5 ≤ *r* < 0.7 is a moderate positive correlation, 0.3 ≤ *r* < 0.5 is a low positive correlation, and *r* < 0.3 is a negligible correlation [23]. Comparing the binding profiles of H3N2 to the H1N1 strains previously published (WSN and the H1N1pdm) produced low genome-wide Pearson correlation coefficients ranging from 0.29 to 0.33 (Table 1). These correlations varied among segments since certain segments had a much stronger similarity when compared to others. For example, segment 2 (*PB1*) exhibited the highest correlation between H3N2 and H1N1pdm viruses (*r* = 0.68, Table 1), and segment 4 (*HA*) had the lowest Pearson correlation (*r* = 0.16, Table 1). These observations are consistent with sequence similarity of these two strains where PB1 nucleotides are 95% similar between Panama and H1N1pdm strains and no similarity is present between the HA nucleotide sequences. In addition, to address whether peak height could bias the Pearson correlation values, we converted the HITS-CLIP data from read counts to a binary value for peak or no peak, 1 and 0 respectively, based on the MACS algorithm. Pearson correlation performed on the binary data resulted in a slight increase in the overall Pearson values but did not alter the trends in observed segments.

Since IAV and IBV vRNA segments differ in length, to compare the pattern of NP-vRNA binding between IAV and IBV strains, we accounted for the difference in size of the segments by aligning the genome sequences and subsequently padding the gaps with the preceding values in the bedGraph file. Surprisingly, when the segments were gap corrected, we found that the Pearson correlation coefficients between IBV and all IAV strains encompassing all eight segments was close to 0 (Table 1). This observation suggests that the IAV and IBV NP binding patterns are unrelated to each other. However, some segments had higher correlation coefficients: IBV segment 8 (*NS*) and WSN had a coefficient of *r* = 0.395 and segment 1 (*PB2*) of IBV and H3N2 had a coefficient of 0.361 while displaying no significant sequence similarity (Figure 2A,H, and Table 1). Overall, we observed that the NP binding pattern over the entire genome of a seasonal H3N2 virus has a low correlation to H1N1 strains while the NP binding pattern of an IBV strain has no correlation to any IAV strain tested.

## 4. Discussion

In this study, we show that seasonal H3N2 and IBV strains display a non-uniform, non-random NP-vRNA binding pattern (Figure 1 and Figure 2), which is similar to H1N1 strains [20,21]. Overall, all strains tested had a similar number of peaks (Figure 3D) and peaks trended to be more G-rich and U-poor compared to non-peaks. These observations provide the first evidence for a nucleotide bias in NP-vRNA binding. In addition, we demonstrate that the frequency of NP-peaks along a vRNA segment does not follow a periodic pattern (Figure 4). Lastly, we compared the NP-binding profile of three IAV, two H1N1, and one H3N2 strain and observed low correlation between strains when all eight segments are concatenated together. Interestingly, the Pearson correlation of NP-binding patterns between segments revealed that some segments were more similar than others, which may be related to sequence similarity or imply a similar relationship during influenza viral RNA assembly.

Efficient incorporation of all eight segments requires segment-specific packaging signals that are known to be in the 3′ and 5′ ends of each vRNA segment [33,34,35,36,37,38,39,40]. Specific regions within the coding sequence of vRNA segments have also been identified using in vitro binding assays [10,11,13,14,41,42]. Many of the regions responsible for vRNA-vRNA interaction were located within the coding region for the avian H5N2 (A/Finch/England/205/91) virus [11,42]. However, these studies were performed on naked RNA without NP. Based on the distinct association of NP to the vRNA segments, the addition of NP might alter the regions of a vRNA segment available for base pairing. A recent study using vRNPs from a H3N2 (A/Udorn/307/72) virus found that segment 2 (*PB1*) between nucleotides 1776 and 2070 interacted with segment 6 (*NA*), which drives co-segregation of these two segments in the virus [43]. In our NP-vRNA binding profile of H3N2, segment 2 (*PB1*) contains two NP-free regions and, interestingly, one of these lies within nucleotides 1776–2070 (Figure 1B, red arrowhead). Therefore, it is an intriguing possibility that the NP-vRNA binding patterns may provide insight into potential vRNA-vRNA interactions.

Segmentation of the influenza virus genome facilitates exchange of vRNPs between co-infecting strains and provides an evolutionary advantage to the virus. Within the human population, H3N2 and H1N1 viruses continuously co-circulate during seasonal epidemics and have previously reassorted to generate a novel H1N2 virus strain [44] that caused a small outbreak during the 2001 to 2002 season. IBV also co-circulates but is evolutionarily divergent and intertypic re-assortment of vRNA segments has never been observed in nature or tissue culture [45]. Interestingly, there is a genetic bias during reassortment where not all 256 (from 2^8^ vRNP choices) possible virus combinations are observed during experimental reassortment studies [46]. Mutations caused by the influenza error-prone polymerase as well as reassortment can promote genetic diversity. However, most re-assortment events are deleterious due to protein incompatibility [47]. Taken together with evidence for intersegmental interactions within the vRNA coding regions, NP binding to vRNA may play a role in potential reassortment events. Variation in influenza NP density along vRNA segments may aid in the coordinated assembly of all eight vRNP segments in an orderly process. Conserved NP-binding patterns between segments from different strains, as we observed in Table 1, may also provide insight into the reassortment potential of certain vRNA segments. Further investigation is needed to determine whether segments with highly similar NP binding patterns are interchangeable and are more likely to be packaged with segments from different strains in a coinfected cell. Expanding on the notion that NP-binding patterns may contribute to vRNA assembly, a re-assortment and the striking differences between IAV and IBV NP-vRNA binding profiles may provide an explanation as to why IAV and IBV are incapable of reassortment.

We demonstrate that NP-vRNA binding profiles of IAV and IBV are non-uniform, which highlights a conservation in the new architecture recently proposed by us and others [20]. Both IAV and IBV encode homologous proteins, which can be distinguished by different protein size, and contain noncoding regions that serve as promoters for replication and transcription [48]. For example, biochemically purified IAV and IBV NP proteins are very similar [49] while the N-terminal domain of IBV is significantly longer than IAV and shares little homology [30,32]. Interestingly, we observed that both IAV and IBV NP proteins have a similar RNA nucleotide bias for binding with preferential association to G-rich and U-poor regions as compared to non-peak regions (Figure 3). It is still unclear what drives NP enrichment at specific sites on the vNRA and further studies are needed to examine the contribution of the RNA sequence and the structure on NP binding.

## Figures and Tables

**Table 1:** Pearson correlation values of NP-vRNA binding profiles for all viruses and segments.

Viral Segments		Virus Comparison					
		H1N1pdm		H3N2		IBV	
		Pearson	*p*-Value	Pearson	*p*-Value	Pearson	*p*-Value
Combined eight segments	WSN	0.42	<0.00001	0.29	<0.00001	−0.01	0.33816
	H1N1pdm			0.33	<0.00001	0.04	<0.00001
	H3N2					−0.02	0.00618
Segment 1 (*PB2*)	WSN	0.40	<0.00001	0.33	<0.00001	−0.07	0.00124
	H1N1pdm			0.20	<0.00001	0.05	0.02013
	H3N2					0.35	<0.00001
Segment 2 (*PB1*)	WSN	0.08	0.00023	0.22	<0.00001	0.14	<0.00001
	H1N1pdm			0.68	<0.00001	0.01	0.73457
	H3N2					0.20	<0.00001
Segment 3 (*PA*)	WSN	0.66	<0.00001	0.65	<0.00001	−0.07	0.00061
	H1N1pdm			0.67	<0.00001	−0.04	0.03624
	H3N2					−0.15	<0.00001
Segment 4 (*HA*)	WSN	0.70	<0.00001	0.17	<0.00001	0.10	2.00 × 10^−5^
	H1N1pdm			0.16	<0.00001	0.30	<0.00001
	H3N2					0.11	<0.00001
Segment 5 (*NP*)	WSN	0.41	<0.00001	0.25	<0.00001	0.22	<0.00001
	H1N1pdm			0.30	<0.00001	0.19	<0.00001
	H3N2					0.01	0.67598
Segment 6 (*NA*)	WSN	0.64	<0.00001	0.20	<0.00001	−0.06	0.0283
	H1N1pdm			0.35	<0.00001	0.01	0.81643
	H3N2					−0.08	0.00274
Segment 7 (*M*)	WSN	0.34	<0.00001	0.70	<0.00001	−0.04	0.20718
	H1N1pdm			0.19	<0.00001	−0.03	0.30731
	H3N2					−0.07	0.01504
Segment 8 (*NS*)	WSN	−0.06	0.03796	0.36	<0.00001	0.29	<0.00001
	H1N1pdm			0.45	<0.00001	−0.10	0.00067
	H3N2					0.13	2.00 × 10^−5^
